# E-Cadherin Is an Accurate Target for Fluorescence-Guided Imaging of Lymph Nodes

**DOI:** 10.3390/cimb48030268

**Published:** 2026-03-03

**Authors:** Kelly A. McGovern, Katherine O. Welch, Jake Mlakar, Ryan Krouse, Michael Brown, Lydia Chen, Kevin Guo, Jeffrey Huang, Edward J. Delikatny, Viktor Gruev, Paul Zhang, Sunil Singhal

**Affiliations:** 1 Department of Thoracic Surgery, Hospital of the University of Pennsylvania, Philadelphia, PA 19104, USA; 2 Department of Radiology, Hospital of the University of Pennsylvania, Philadelphia, PA 19104, USA; 3 Department of Engineering and Computer Engineering, University of Illinois at Urbana-Champaign, 306 N Wright St, Urbana, IL 61801, USA; 4 Department of Pathology and Laboratory Medicine, Hospital of the University of Pennsylvania, Philadelphia, PA 19104, USA

**Keywords:** intraoperative molecular imaging, fluorescence-guided imaging, biomarker, lung cancer

## Abstract

Lymph node (LN) dissection is a necessary part of every oncologic surgery in order to provide important information for staging, predicting prognosis and improving survival. To do this, surgical oncologists strive to localize and dissect every pathologically positive LN while avoiding the increased morbidity of removing true negative LNs. The goal is to develop an imaging method to distinguish positive and negative LNs, but a specific biomarker is missing. Thus, our aim is to identify a reliable imaging marker for identifying LNs with lung cancer cells. After screening many epithelial markers, we identified E-cadherin, a membrane protein normally expressed in epithelial cells, including in the lung. To follow up on our potential target, we performed immunofluorescence staining on 48 human LNs with a conjugated anti-E-cadherin monoclonal antibody. Fluorescence was significantly higher in LNs with metastasis, as shown in 48 positive LNs from patients with resected primary lung cancer. There was high fluorescence in both hilar and mediastinal LNs, and in all primary tumor histologies. E-cadherin may be useful for the surgical oncologist for targeted imaging technologies for selecting positive LNs from lung cancer.

## 1. Introduction

Lymph node (LN) excision is essential in oncologic surgery to provide important information in staging cancers, predicting prognosis and improving survival [1,2,3]. The process of harvesting LNs during cancer surgery varies for different cancer types, such as lung, breast, thyroid, colon, and gastric cancers.

In lung cancer surgery, some surgeons opt to perform a lobe-specific approach to LN dissection, resecting only the LNs contiguous to the resected lung lobe. This method may result in missed LNs harboring cancer cells (“positive LNs”) in 6% of patients because positive LNs may be present in non-contiguous regions [4]. This can result in inaccurate staging, failure to offer adjuvant treatment, and decreased survival rates. The most recent guidelines from the European Society of Thoracic Surgeons (ESTS) recommend a systematic LN dissection, including dissection of at least 3 mediastinal LN stations and requiring stations 7 (subcarinal), 10 (hilar), and 11 (interlobular) [5,6]. These differences in practice highlight a major challenge for surgical oncologists to intraoperatively identify LNs harboring cancer cells.

Ideally, a surgical oncologist would be able to localize and dissect all positive LNs while avoiding the increased morbidity of removing true negative LNs or performing a larger systematic dissection. However, at present, this is exceedingly challenging because of the similar color, shape, and consistency of positive and negative LNs. The current guidelines recommend a non-specific aggressive dissection of all LN stations to reduce false negatives, but this may increase the morbidity to the patient because of potential damage to lymphatic channels, nerves, and blood vessels.

Multiple imaging techniques have emerged to improve targeted surgical procedures. Many of these involve targeted contrast agents such as targeted PET tracers preoperatively and targeted fluorescent contrast agents intraoperatively to selectively accumulate in tumors [7,8]. Despite these advancements, no currently available targeted contrast agent effectively distinguishes LNs with cancer cells from normal LNs.

Our goal was to identify a reliable marker for imaging to denote a positive from a negative LN. After a broad screen of many biomarkers for NSCLC, we identified E-cadherin (CDH1, CD324) as a possible candidate for differentiating positive and negative LNs. E-cadherin is a transmembrane protein that contributes to tissue homeostasis via calcium-dependent signaling and functions in cell adhesion and cell junction formation. It is physiologically expressed in epithelial tissues, including the lung, and it is not present on normal LNs. It has been shown to be upregulated in rapidly growing epithelial tumors and acts primarily to prevent tumor progression due to its adhesive function, preventing tumor spread [9,10,11,12].

E-cadherin is an accepted marker for lung cancer, and we hypothesized that E-cadherin would be a reasonable imaging target to identify LNs with metastatic lung cancer cells and differentiate them from LNs without metastatic lung cancer cells. In this study, we aim to characterize the imaging characteristics when targeting E-cadherin in positive LNs compared to negative LNs in patients with lung cancer.

## 2. Methods

### 2.1. Assessing E-Cadherin Expression in Lung Cancer Metastases by Immunohistochemistry

Tissue specimens were obtained from 48 LNs with pulmonary metastases from 18 patients who underwent lung cancer surgeries at the Hospital of the University of Pennsylvania. Written informed consent for tissue collection was obtained for all subjects, and the study was conducted in accordance with the institutional review board of the University of Pennsylvania. Patients were excluded if they had a primary tumor histology other than lung cancer or did not have positive LNs found on postoperative pathologic assessment. All patients were operated on at the Department of Thoracic Surgery, Hospital of the University of Pennsylvania, and were given written informed consent. The study was conducted in accordance with and approved by the university’s Institutional Review Board.

Samples were prepared and immunostained for E-cadherin using anti-E-cadherin monoclonal antibody (mAb) (ECM Biosciences, Aurora, CO, USA; catalog number CM1681; 1:500 dilution) applied to formalin-fixed, paraffin-embedded sections. Next, 5-micron thick sections underwent deparaffinization, rehydration, and washings in xylene, graded alcohols, and distilled water. The sections were then placed in 10 mM citrate buffer at pH 6 with subsequent microwave antigen retrieval. The antigen-antibody reaction was visualized using avidin–biotin–peroxidase complex. Diaminobenzidine was used as the chromogen, and the slides were counterstained with hematoxylin. Positive LNs versus negative LNs were distinguished by pathological assessment of resected specimens as evaluated by a board-certified pulmonary pathologist.

### 2.2. Assessing E-Cadherin Expression in Human Lung Cancer Metastases by Immunofluorescence

The unstained slides from patient positive and negative LN samples were then evaluated for fluorescence with an anti-E-cadherin mAb conjugated to AlexaFluor647 (excitation 650 nm, emission 671 nm) (Waltham, MA, USA; catalog number ab194982). The sections underwent deparaffinization, rehydration, and washings in xylene, graded alcohols, and distilled water. The sections were placed in 10 mM citrate buffer at pH 6 with a subsequent microwave antigen retrieval procedure. The slides were incubated with the mAb at a 1:200 dilution. Slides were mounted with ProLong Gold Antifade Reagent containing the nuclear dye 4′,6-diamidino-2-phenylindole (DAPI) (Fisher Scientific, Waltham, MA, USA). Fluorescence was imaged with a Leica DM6 B fluorescence microscope (Leica Microsystems, Wetzlar, Germany) with a Cy5 filter, as recommended for AlexaFluor647 conjugates.

### 2.3. Post Hoc Image Analysis

Post hoc image analysis was conducted using ImageJ (Version 1.54) [13]. Using H&E samples, the proportion of the area containing tumor cells compared to the overall area of the LN was calculated to obtain the percent of cancer cells in the LN, or percent tumor burden. On immunofluorescence samples, after correcting for distant background fluorescence, the mean fluorescence intensity (MFI) of the tumor and the background was measured to calculate a tumor-to-background ratio (SBR) for each sample. An SBR > 2 was designated as the cutoff for sufficient fluorescent contrast to delineate tumor from background within each sample. The positive LN signal was compared to the negative LN signal by comparing the MFI of the two samples, termed the tumor-to-normal ratio (TNR). To maintain consistency, all fluorescence values below 1 were adjusted to a minimum value of 1.

### 2.4. Chart Review and Subgroup Analyses

Under a University of Pennsylvania IRB-approved protocol, a chart review was performed from the electronic medical record. For patients with LNs with pulmonary metastasis, information was collected, including patient and tumor characteristics. Subsets of the positive and negative LN groups were analyzed to determine if E-cadherin positivity correlated with clinical characteristics. Anatomic proximity of positive LNs was analyzed to determine correlation with E-cadherin expression. Primary tumor histology was also analyzed to determine correlation with E-cadherin expression. Event-free survival was captured, with events including recurrence, relapse, or death.

### 2.5. Statistical Analysis

Statistical analysis was performed using GraphPad Prism 8 (GraphPad Software, San Diego, CA, USA). Data analysis is stratified by positive LNs compared to negative LNs. Patient and tumor characteristics are summarized using descriptive statistics or proportions. Between-group analysis for tumor characteristics is performed using paired *t*-tests. Log-rank test was performed for event-free survival. *p*-Values less than 0.05 were considered statistically significant.

## 3. Results

### 3.1. Metastatic LNs Fluoresce with Anti-E-Cadherin-AlexaFluor647

Based on encouraging preclinical data, we aimed to confirm our findings in human tissues from patients with resectable non-small cell lung cancer. We identified a set of 18 patients who underwent pulmonary resection for lung cancer. Table 1 describes characteristics of the included patients. Patients had a mean age of 57.9 ± 11.3, and all patients had pathologic N1 or N2 stage disease on postoperative pathologic assessment. In the 18 patients, we obtained a total of 48 lymph nodes (LNs) that harbored cancer cells and 210 normal LNs without cancer cells. Each sample underwent anti-E-cadherin monoclonal antibody (mAb) immunofluorescence (IF) staining and was reviewed by a board-certified lung pathologist.

**Figure 1:**
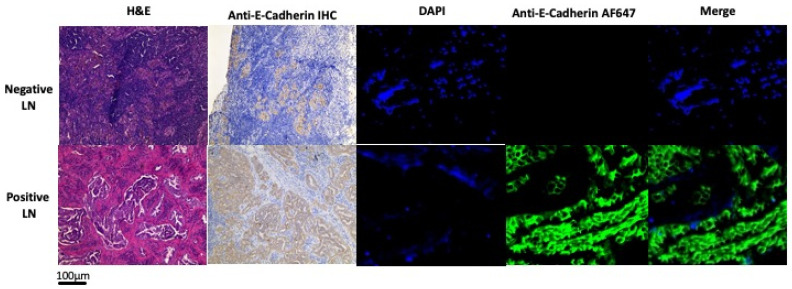
Representative immunohistochemistry and immunofluorescence images in negative lymph nodes (**upper row**) and positive lymph nodes (**bottom row**), showing increased fluorescence consistent with the presence of tumor cells in immunohistochemistry and hematoxylin and eosin (H&E) staining (LN, lymph node; DAPI, 4′,6-diamidino-2-phenylindole; IHC, immunohistochemistry; AF647, AlexaFluor-647).

Of the 48 LNs that harbored cancer cells, 100% of them had fluorescence to some degree, which was indicative of E-cadherin expression. As shown in Figure 1, the areas of E-cadherin expression in the positive LNs were consistent with areas of cancer cells in the LN.

### 3.2. Quantification of E-Cadherin Fluorescence in Pulmonary Metastases in LNs

**Figure 2:**
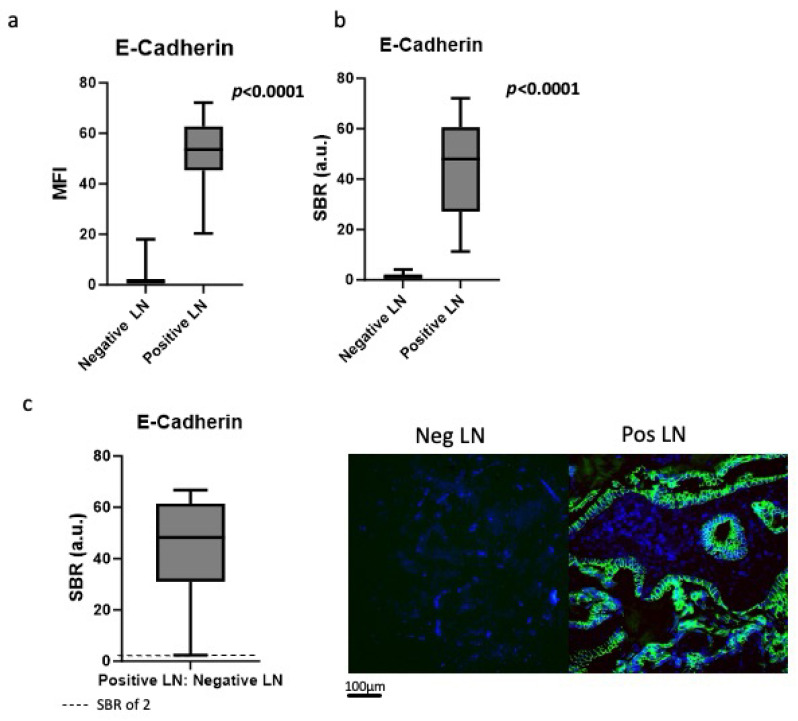
Quantification of E-cadherin targeted fluorescence. (**a**) MFI of positive LNs was 53.0 (IQR 45.3–62.8), significantly higher than in the negative LN specimens, which had MFI of 2.7 (IQR 1.0–2.1) (*p* < 0.0001). (**b**) The SBR was significantly higher in the positive LNs compared to the negative LNs 34.4 vs. 1.5, *p* < 0.0001). (**c**) When the MFI of the positive LNs were compared to the negative LNs from the same patient, the resulting TNR was 45.3 (IQR 31.1–61.4).

Next, our goal was to quantify the E-cadherin fluorescence in positive versus negative LNs. The mean fluorescence intensity (MFI) was 53.0 (IQR 45.3–62.8) in positive LN specimens, significantly higher than the 210 negative LN specimens, which had an MFI of 2.7 (IQR 1.0–2.1) (*p* < 0.0001) (Figure 2a). The fluorescence was confirmed to correlate with areas of cancer cells within the LNs.

Similarly, the Signal-to-Background Ratio (SBR) was significantly higher in the positive LNs compared to the negative LNs (34.4 vs. 1.5, *p* < 0.0001) (Figure 2b). When the MFI of the positive LNs was compared to the negative LNs from the same patient, the resulting TNR was 45.3 (IQR 31.1–61.4), revealing a significant intra-patient difference between negative and positive LNs (Figure 2c).

### 3.3. E-Cadherin Fluoresces in Both Hilar and Mediastinal Lymph Node Metastases

**Figure 3:**
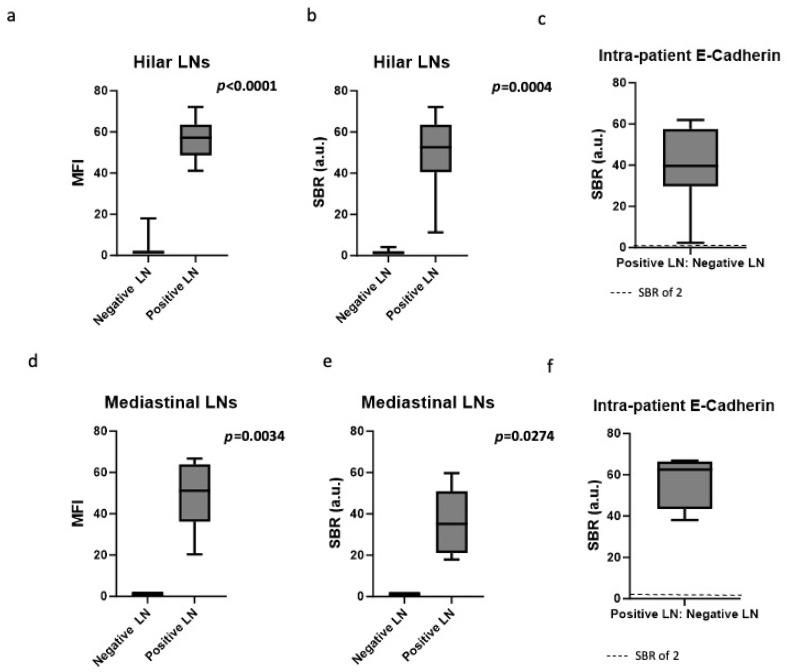
Quantification of E-cadherin-targeted fluorescence in proximal (hilar) vs. distant (mediastinal) LNs. (**a**) In hilar LNs, the MFI was significantly higher in positive LNs compared to negative LNs (56.4 vs. 3.6, *p* < 0.0001). (**b**) The SBR was significantly higher in the positive hilar LNs compared to the negative hilar LNs (49.7 vs. 1.7, *p* = 0.0004). (**c**) When the MFI of the positive hilar LNs were compared to the negative LNs from the same patient, the resulting TNR was 39.2 (IQR 29.8–57.5), revealing a clinically significant intra-patient difference. (**d**) In mediastinal LNs, MFI was significantly higher in positive LNs compared to negative LNs (48.5 vs. 1.0, *p* = 0.0034). (**e**) Similarly, in mediastinal LNs, SBR was higher in positive LNs compared to negative LNs (36.1 vs. 1.0, *p* = 0.0274). (**f**) The intra-patient MFI ratio (TNR) was 57.5 (IQR 43.5–66.5).

We next sought to determine if there was any difference in E-cadherin fluorescence in positive LNs that are spatially close to or distant from the primary pulmonary malignancy. We used hilar LNs as a surrogate for a “close” LN, as they are anatomically within the same lung lobe as the cancer. We used mediastinal LNs as a surrogate for “distant” LNs, as they are no longer incorporated within the pleural lining of the lung. Of 24 hilar LNs, the MFI was significantly higher in the positive LNs compared to the negative LNs (56.4 vs. 3.6, *p* < 0.0001) (Figure 3a). Similarly, the SBR was significantly higher in the positive LNs compared to the negative LNs (49.7 vs. 1.7, *p* = 0.0004) (Figure 3b). When the MFI of the positive hilar LNs were compared to the negative LNs from the same patient, a surrogate for how SBR would be measured in vivo, the resulting ratio was 39.2 (IQR 29.8–57.5), revealing a significant intra-patient difference between positive and negative hilar LNs (Figure 3c).

Of 25 mediastinal LNs, the fluorescence as measured by MFI (48.5 vs. 1.0, *p* = 0.0034) (Figure 3d) and SBR (36.1 vs. 1.0, *p* = 0.0274) (Figure 3e) was higher in the positive LNs compared to the negative LNs. When the MFI of the positive mediastinal LNs was compared to the negative LNs from the same patient, the resulting TNR was 57.5 (IQR 43.5–66.5), again revealing a significant intra-patient difference between positive and negative mediastinal LNs (Figure 3f).

### 3.4. E-Cadherin Fluoresces in Lymph Node Metastases from Different Primary Histologic Types

**Figure 4:**
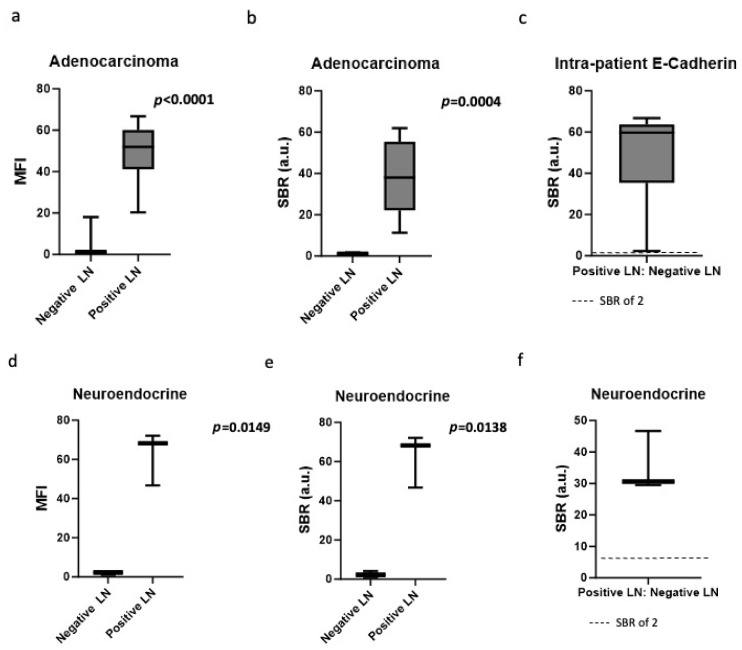
Quantification of E-cadherin-targeted fluorescence in positive LNs with different primary tumor histologies. (**a**) MFIs of positive LNs from adenocarcinoma primary tumors were significantly different compared to negative LNs (51.0 vs. 3.0, *p* < 0.0001). (**b**) SBR was significantly different in positive LNs from adenocarcinoma primary tumors compared to negative LNs (39.9 vs. 1.2, *p* = 0.0004). (**c**) The intra-patient MFI ratio was 48.5 (IQR 35.3–63.7), revealing a significant intra-patient difference between positive and negative LNs. (**d**) Of LNs from primary neuroendocrine tumors, the MFI (62.4 vs. 1.9, *p* = 0.0149) (**e**) and SBR (62.4 vs. 2.5, *p* = 0.0138) were higher in positive LNs. (**f**) The intra-patient MFI ratio (TNR) was 36.6 (IQR 29.5–46.7), revealing a large intra-patient difference.

We next hypothesized that E-cadherin expression may only correlate with certain histological subtypes of non-small cell lung cancer. Primary tumor histology was analyzed for correlation with E-cadherin-targeted fluorescence in positive LNs, including primary adenocarcinoma tumors (n = 42) and primary neuroendocrine tumors (n = 7). Of LNs from primary adenocarcinoma tumors, the MFI (51.0 vs. 3.0, *p* < 0.0001) (Figure 4a) and SBR (39.9 vs. 1.2, *p* = 0.0004) (Figure 4b) were significantly higher in the positive LNs compared to the negative LNs. When the MFI of the positive hilar LNs was compared to the negative LNs from the same patient, the TNR was 48.5 (IQR 35.3–63.7), revealing a significant intra-patient difference between positive and negative LNs (Figure 4c).

Of LNs from primary neuroendocrine tumors, the fluorescence as measured by MFI (62.4 vs. 1.9, *p* = 0.0149) (Figure 4d) and SBR (62.4 vs. 2.5, *p* = 0.0138) (Figure 4e) was higher in the positive LNs compared to the negative LNs, although the sample size was small. When the MFI of the positive hilar LNs was compared to the MFI of the negative LNs from the same patient, the resulting TNR was 36.6 (IQR 29.5–46.7), again revealing a significant intra-patient difference for in vivo application (Figure 4f).

### 3.5. E-Cadherin Fluorescence Is Measurable Even at Low Tumor Burden in Lymph Nodes

**Figure 5:**
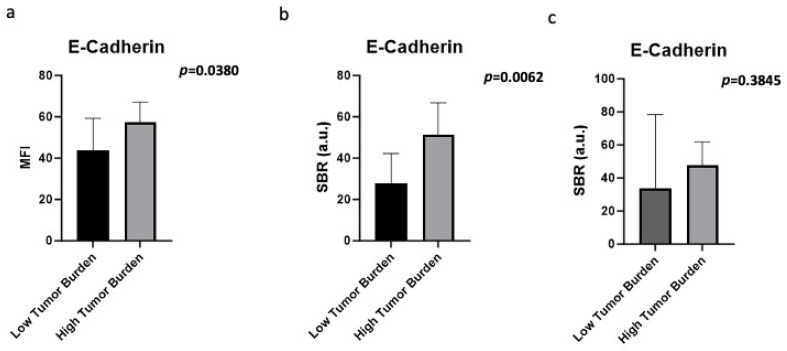
E-cadherin- targeted fluorescence in relation to LN tumor burden. (**a**) There was no difference in MFI between positive LNs with micrometastases compared to those with macrometastases. (**b**) There was no difference in MFI between positive LNs with micrometastases compared to those with macrometastases. (**c**) There was no difference in TNR between positive LNs with micrometastases compared to those with macrometastases, revealing high sensitivity for low tumor burden.

Since lung cancer recurrence may often be secondary to micrometastases in LNs, our next goal was to assess whether E-cadherin-targeted fluorescence would be present in LNs with low tumor burden. We used tumor burden in an LN of less than 5% as a surrogate for micrometastases or “low tumor burden”, and tumor burden of greater than or equal to 5% as a surrogate for macrometastasis or “high tumor burden”. MFI was higher in LNs with high tumor burden compared to low tumor burden (*p* = 0.0380) (Figure 5a). Similarly, SBR was higher in LNs with high tumor burden compared to low tumor burden (*p* = 0.0062); however, the mean SBR was over 2, the clinically appreciable level of fluorescence, even in the low tumor burden LNs (Figure 5b). When the MFIs of positive LNs were compared to the MFIs of negative LNs from the same patient, acting as an intra-patient normalization, the TNRs were similar between the low tumor burden and high tumor burden LNs, again showing clinical applicability of targeting E-cadherin for fluorescence in clinical scenarios (*p* = 0.3845) (Figure 5c).

### 3.6. Event-Free Survival

**Figure 6:**
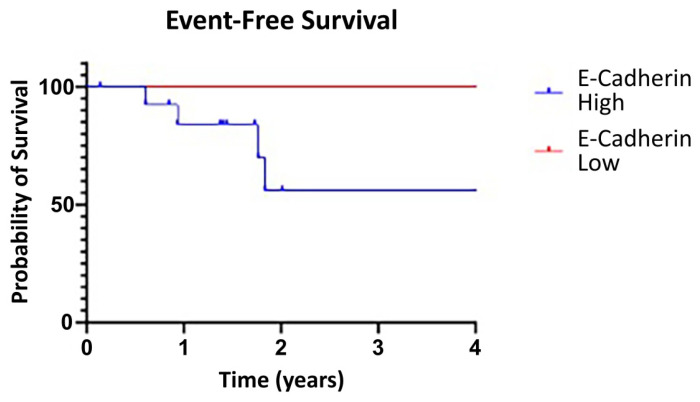
Event-free survival evaluated by MFI, as grouped by high E-cadherin (upper three quartiles) and low E-cadherin (lower quartile), revealed no significant difference.

Event-free survival (EFS) was evaluated to see if MFI correlated with clinical outcomes. Patients were grouped by high E-cadherin (upper three quartiles of MFI) and low E-cadherin (lower quartile of MFI), and a log-rank test was performed for EFS. There was no significant difference in EFS between the two groups, although there was clear separation between the EFS of the E-cadherin-high and E-cadherin-low groups at four years (Figure 6). Notably, our study was not powered to evaluate survival, but given these results, with adequate powering for survival, high fluorescence from targeting E-cadherin would likely correspond to worse clinical outcomes.

## 4. Discussion

In this study, we discovered that using E-cadherin as a target for a targeted imaging probe for PET or optical imaging of lymph nodes (LNs). We found that E-cadherin was equally effective at labeling hilar and mediastinal LNs, and there was no difference in the E-cadherin fluorescence across different primary tumor histologies. Our findings demonstrate that targeting E-cadherin with a fluorescent probe allows for reliable labeling of tumor cells in LNs with minimal background fluorescence.

As an epithelial adhesion molecule, E-cadherin expression in lung cancer has been well described. Increased E-cadherin is associated with initial tumor growth, and subsequent reduced E-cadherin expression is associated with the epithelial-mesenchymal transition and metastasis [10,14]. A study of 111 lung cancer patients found that E-cadherin expression and lymph node stage were significantly inversely correlated, and concluded that decreased E-cadherin expression was associated with tumor dedifferentiation, increased LN metastasis and worse survival [15]. In 480 patients with invasive ductal breast carcinoma, a significant correlation between the loss of E-cadherin expression and LN metastasis was found [11]. Similarly, among 77 patients with bladder transitional cell carcinoma, loss of normal membrane E-cadherin expression was found in the majority of patients in both positive LNs and in primary tumors [16]. Although these studies emphasized decreased E-cadherin expression compared to the primary tumor, we underscore with our results that E-cadherin expression persists even after metastasis, as all of our pathologically positive LNs exhibited some level of E-cadherin fluorescence. This suggests that, even if the E-cadherin expression is slightly decreased in the positive LNs compared to the primary lung cancer, E-cadherin still presents a sensitive target for optical or nuclear imaging for positive LN identification.

Occult N2 disease and skip metastases, in which the cancer goes to a farther lymph node skipping over a close LN, continue to be major challenges for thoracic surgeons. There is conflicting data on the prognosis of skip N2 disease, which highlights the need for effective LN dissections to ensure patients receive necessary adjuvant treatments [5,6,17,18]. Currently, cancer surgeons rely on visual and tactile cues to identify irregular nodules, and frozen section is typically used to confirm pathologic diagnosis; however, this may be time-consuming and variable in accuracy. These methods, although helpful for the primary tumor, often do not provide the same utility for LN identification. Frozen section for LNs is not reliable in lung cancer due to the possibility of skip metastases [19,20], and visual and tactile cues will not detect microscopic nodal metastases.

One potential application is targeted optical imaging, also called intraoperative molecular imaging or fluorescence-guided surgery, which utilizes injected fluorescent contrast agents that target cancer cells to improve intraoperative localization and margin assessment [7,21,22]. IMI has shown success in enhancing resection of many solid tumors, including lung, glioma, breast, and head and neck cancers [21,22,23,24,25,26,27,28]. Currently used molecular targets include cathepsins, annexins, and folate receptor [21,23,28,29,30,31]. Although these have been useful in accurately detecting primary tumors, there is not yet a target for LN metastases.

Cathepsins are lysosomal cysteine proteases involved in protein degradation, and they are found to be overexpressed by tumor cells and tumor-associated macrophages (TAMs). VGT-309 is an activity-based probe that is activated by cathepsins, fluorescing where there is high cathepsin activity, such as the tumor microenvironment [28,29,30]. Non-cancerous inflammatory processes may cause increased cathepsin expression and potentially result in a false positive. Therefore, inflammatory or reactive LNs may have false positives without having cancer cells with this imaging agent.

Pafolacianine’s target is the folate receptor alpha (FRα), a cell surface glycoprotein that is overexpressed in many cancers, including non-small cell lung cancer (NSCLC). FRα expression is low in normal lung tissues, making it an excellent molecular target for IMI of NSCLC [21,23,25]. Although Pafolacianine is targeted to FRα, it may also bind to folate receptor beta (FRβ), another isoform of the folate receptor. This hinders IMI of LNs with cancer cells because macrophages express FRβ, and this expression is particularly prominent in TAMs. Therefore, there would be significant false-positive fluorescence in LNs due to FRβ from TAMs.

E-cadherin as a biomarker for LN metastases may present an opportunity for use in IMI for precision LN dissection. Although existing literature states that there is downregulation of E-cadherin leading to the epithelial-mesenchymal transition and subsequent metastasis, there remains significant expression of E-cadherin in LN metastases compared to normal LNs, and compared to normal epithelial tissue. E-cadherin is a promising marker of micrometastases to LNs at occult or subclinical levels. Utilizing this information as an adjunct to stage positive LNs that would have otherwise been undetected may allow for more precise postoperative treatment and fewer cancer recurrences secondary to missed nodal metastases. Because there is minimal background fluorescence with E-cadherin in negative LNs, E-cadherin is a highly specific and sensitive target. Although E-cadherin is a known epithelial tumor marker, this is the first study looking at E-cadherin as a target for IMI of LNs with cancer cells.

These results with E-cadherin are promising, but limitations are acknowledged. Given the retrospective nature, small sample size, and the utilization of formalin-fixed paraffin-embedded tissues, in vivo imaging could not be confirmed with the tissue available. Despite this, we used a reliable mAb and achieved consistent results across all samples. Additionally, we believe the results to be reproducible given the standardized methodology with antibody-based immunofluorescence. Larger studies, including animal models or human intraoperative trials, may assist with validation.

In summary, E-cadherin is a highly promising solution for IMI in targeted LN dissection in lung cancer surgeries. E-cadherin showed strong specificity and low background fluorescence in all LN specimens, even at low tumor burdens in both hilar and mediastinal LNs and in different primary tumor histologies. E-cadherin may be useful for thoracic surgeons for intraoperative detection of positive LNs from epithelial cancers, including lung cancer, and this warrants further investigation with in vivo studies.

## Figures and Tables

**Table 1:** Patient characteristics.

Variables	N (%)
Age	57.9 ± 11.3
Sex	
Male	7 (38.9)
Female	11 (61.1)
Location	
Hilar	24 (49.0)
Mediastinal	25 (51.0)
Histology	
Adenocarcinoma	42 (85.7)
Neuroendocrine	7 (14.3)
Pathologic N Stage	
N0	0 (0)
N1	24 (49.0)
N2	25 (51.0)
N3	0 (0)

## Data Availability

The original contributions presented in this study are included in the article. Further inquiries can be directed to the corresponding author.
